# Effect of staurosporine on the mobility and invasiveness of lung adenocarcinoma A549 cells: an in vitro study

**DOI:** 10.1186/1471-2407-9-174

**Published:** 2009-06-08

**Authors:** Yanyan Wang, Hongfa Yang, Hongbin Liu, Ji Huang, Xingfu Song

**Affiliations:** 1 Department of Pharmacology, The First College of Clinical Medical Science, China Three Gorges University, 183 Yiling Road, Yichang 443003, PR China; 2 Department of Pharmacology, Yichang Central People's Hospital, 183 Yiling Road, Yichang 443003, PR China

## Abstract

**Background:**

Lung cancer is one of the most malignant tumors, representing a significant threat to human health. Lung cancer patients often exhibit tumor cell invasion and metastasis before diagnosis which often render current treatments ineffective. Here, we investigated the effect of staurosporine, a potent protein kinase C (PKC) inhibitor on the mobility and invasiveness of human lung adenocarcinoma A549 cells.

**Methods:**

All experiments were conducted using human lung adenocarcinoma A549 cells that were either untreated or treated with 1 nmol/L, 10 nmol/L, or 100 nmol/L staurosporine. Electron microscopy analyses were performed to study ultrastructural differences between untreated A549 cells and A549 cells treated with staurosporine. The effect of staurosporine on the mobility and invasiveness of A549 was tested using Transwell chambers. Western blot analyses were performed to study the effect of staurosporine on the levels of PKC-α, integrin β1, E-cadherin, and LnR. Changes in MMP-9 and uPA levels were identified by fluorescence microscopy.

**Results:**

We demonstrated that treatment of A549 cells with staurosporine caused alterations in the cell shape and morphology. Untreated cells were primarily short spindle- and triangle-shaped in contrast to staurosporine treated cells which were retracted and round-shaped. The latter showed signs of apoptosis, including vacuole fragmentation, chromatin degeneration, and a decrease in the number of microvilli at the surface of the cells. The A549 cell adhesion, mobility, and invasiveness significantly decreased with higher staurosporine concentrations. E-cadherin, integrin β1, and LnR levels changed by a factor of 1.5, 0.74, and 0.73, respectively compared to untreated cells. In addition, the levels of MMP-9 and uPA decreased in cells treated with staurosporine.

**Conclusion:**

In summary, this study demonstrates that staurosporine inhibits cell adhesion, mobility, and invasion of A549 cells. The staurosporine-mediated inhibition of PKC-α, induction of E-Cad expression, and decreased integrin β1, LnR, MMP-9, and uPA levels could all possibly contribute to this biological process. These results represent a significant step forward in the ongoing effort to understand the development of lung carcinoma and to design novel strategies to inhibit metastasis of the tumor by targeting the cell-adhesion, mobility and invasion of tumor cells.

## Background

Lung cancer is one of the most malignant tumors and represents a significant threat to human health. The strong invasive and metastatic characteristics of lung tumor cells are responsible for its relatively high malignancy. Indeed, patients with lung cancer often exhibit tumor cell invasion and metastasis before diagnosis which renders current treatments, including surgery, radiotherapy, and chemotherapy ineffective. Typically, 85% of lung cancer patients die within a period of 5 years after diagnosis. Therefore, studying the molecular basis of lung cancer cell invasion and metastasis is crucial in order to design new therapeutic agents that prevent or slow lung cancer cell invasion and metastasis.

Tumor cell invasion and metastasis are inter-related processes associated with adhesion of tumor cells, hydrolysis of the extracellular matrix, cell mobility, and the regulation and expression of metastasis related genes. Protein kinases are involved in all of the above mentioned processes. Protein kinase C (PKC), an important member of the protein kinase family, is a calcium-activated and phospholipid-dependent serine/threonine protein kinase. PKC phosphorylates a number of substrates to mediate a series of physiological responses, including cell growth, proliferation, differentiation, apoptosis, and mobility [1-4]. Furthermore, PKC is also important for the maintenance of normal physiological functions of cells [5]. It has been demonstrated that the PKC level, which is closely related to the invasiveness and metastasis of tumor cells, is enhanced in some tumors [6]. It was recently shown that the level of PKC-α is significantly higher in lung cancer tissue when compared to healthy lung tissue, and its trafficking to the cell membrane and the nuclei is also increased significantly [7,8]. Moreover, examination of clinical samples showed that the levels of PKC-α protein correlated with lung cancer TNM staging. Higher PKC-α levels were seen in more advanced stages with higher metastatic and invasive capabilities. It has been suggested that the over-expression of PKC-α and its cytomembrane transportation play a role in regulating the progress and metastasis of lung cancer cells [7,8]

Staurosporine is a potent inhibitor of PKC and many other kinases, including the tyrosine protein kinase. It blocks the transfer of the phosphate ester from DNA to the activated tyrosine sites and directly inhibits the activity of topoisomerase II. It has been reported [9,10] that staurosporine can induce apoptosis of a variety of cells, including cardiac cells, oral squamous cell carcinoma cells, and fibroblasts. Therefore, staurosporine is widely used to study apoptosis and has become one of the most promising anti-cancer drugs. Although staurosporine has been well studied in the context of apoptosis in cancer cells, not much information is available on the role of staurosporine on cell adhesion, mobility and invasion in lung cancer in the context of tumor metastasis.

Based on the above information, we hypothesized that staurosporine-mediated inhibition of PKC could affect the invasive and metastatic capabilities of lung tumor cells, exerting its anti-tumor function through mechanisms other than the induction of apoptosis. In this study, we treated human lung adenocarcinoma A549 cells with staurosporine and investigated the relationship between staurosporine treatment and tumor cell adhesion, mobility, and invasiveness. We also studied the effect of staurosporine on the levels of adhesion molecules, including integrin β1, E-cadherin, LnR, and on the levels of proteolytic enzymes MMP-9 and uPA. The feasibility of using staurosporine as a compound that blocks invasion and metastasis of lung tumor cells was explored.

## Methods

### Cell line and experimental grouping

Human lung adenocarcinoma cell line A549 was obtained from the American Typical Culture Center (ATCC), passaged and preserved at the Chinese Culture Center, Wuhan University (Wuhan, China). The cells were cultured in RPMI 1640 medium (free of phenol red) containing 10% FCS and maintained at 37°C in 5% CO_2_. Staurosporine (Sigma, St. Louis, MO) was dissolved in dimethyl sulfoxide (DMSO) to a final concentration of 1 nmol/L, 10 nmol/L, and 100 nmol/L [6]. For the control experiments, cells were incubated in RPMI 1640 medium containing 0.1 ‰ DMSO and 10% FCS (without staurosporine).

### Adhesion experiment

96-well plates were coated with Matrigel (Department of Cell Biology, School of Medicine, Peking University, Beijing, China) and air dried in a laminar hood overnight. The wells were blocked with 2% bovine serum albumin (BSA; 50 μL/well) and incubated at 37°C for 2 h. A549 cells were inoculated into the 96-well plate at a concentration of 1 × 10^3 ^cells/well. The medium was discarded after 2 h, and wells were washed with 200 μL phosphate-buffered saline (PBS) to remove unattached cells. Subsequently, 12 μL MTT (Sigma; 5.0 mg/mL) was added into each well and the samples were incubated at 37°C for 4 h. Isopropanol (100 μL) was added into each well and the samples were mixed well. The purplish-blue crystals were dissolved at room temperature. The absorbance (A) at λ = 540 nm was read on the spectra Max Plus 384 Molecular Devices (Sunnyvale, CA) and the data were analyzed. Each group consisted of four duplicates and the cell adhesion inhibition rate was calculated using the following equation:

Inhibition rate = (A of control group - A of drug administration group)/A of control group × 100%.

### Cell mobility experiment

Transwell chambers (Costar, Bethesda, MD) were used in the cell mobility experiments. Cells were inoculated into the upper compartment of the Transwell chambers at a concentration of 1 × 10^5 ^cells/mL and 100 μL/well. The medium for the experimental and control group was added into the lower compartment of the Transwell chambers (500 μL/well). The cells were cultured at 37°C for 10 h. Cells that did not penetrate the polycarbonate membrane at the bottom of the chamber were wiped off with cotton stickers. The membrane was removed and fixed with methanol and stained with Hematoxylin & Eosin. Five vision fields were randomly selected under a BX50 microscope (Olympus, Tokyo, Japan) and the number of cells that penetrated the membrane was counted. Each group consisted of four duplicates. The mobility inhibition rate was calculated using the following equation:

Mobility inhibition rate = (number of cells in control group that penetrated the membrane - number of cells in the staurosporine group that penetrated the membrane)/number of cells in control group that penetrated the membrane × 100%.

### Cell invasion experiment

Transwell chambers were used to determine the cell invasiveness. The membrane at the bottom of the Transwell chamber was evenly coated with 50 μL Matrigel and air dried in a laminar hood overnight. The samples were blocked with 2% BSA, 50 μL/well and incubated at 37°C for 2 h and were then rinsed with PBS buffer. Cells were inoculated into the upper compartment of the Transwell chambers at a concentration of 2 × 10^5 ^cells/mL and 100 μL/well. The medium for the experimental and control group was added into the lower compartment of the Transwell chamber (500 μL/wel)l. The cells were cultured at 37°C for 48 h. Cells that did not penetrate the polycarbonate membrane at the bottom of the chamber were wiped off with cotton stickers. The membrane was removed and fixed with methanol and stained with HE. Five vision fields were selected randomly under the BX50 microscope (Olympus), and the number of cells that penetrated the membrane was counted. Each group consisted of duplicates. The invasion inhibition rate was calculated using the following equation:

Invasion inhibition rate = (number of cells in control group that penetrated the membrane - number of cells in staurosporine group that penetrated the membrane)/number of cells in control group that penetrated the membrane × 100%.

### MTT assay

A549 cells were inoculated in 96 well plates at 1 × 10^5 ^cells/well in phenol-red free RPMI 1640 medium. After drug treatment, 12 μL MTT (5.0 mg/mL) was added into each well and the cells were incubated at°C for 4 h. After incubation, 100 μL isopropanol was added into each well and the samples were mixed well. The purplish-blue crystals were dissolved at room temperature. The absorbance (A) at λ = 540 nm was read on the spectra Max Plus 384

### FACS apoptosis assay

Cells were collected and digested with 0.25% trypsin and 0.02% EDTA (1:1) at 37°C for 3–4 min. Cells were pipetted gently and then collected by centrifugation at 1000 rpm for 5 min. They were then washed twice with cold PBS (0.01 M pH 7.4) and resuspended in the residual PBS. After adding 1 mL prechilled (-20°C) 80% ethanol, the cells were stored at -20°C over night. After washing twice with PBS, 60–80 μL RNAase (1 mg/mL) was added and cells incubated at 37°C for 30 min. After chilling on ice for 2 min, PI solution (100 mg/L PI, 0.1% TritonX-100) was added and the sample was incubated in the dark for 30 min. The cell cycle distribution and apoptosis were analyzed by flow cytometry (FACSCalibur, Becton Dickinson, Franklin Lakes, NJ USA). Data acquired by CELLQuest was analyzed using the software ModFit LT that came with the machine.

### Transmission electron microscope

A549 cells cultured for 24 h, were collected from the culture flask wall with a cell shovel and prepared as a cell suspension of 1 × 10^6 ^cells/ml. The samples were centrifuged at 1000 rpm/min for 10 min, and the supernatant was discarded. Samples were rinsed with 0.01 M PBS twice. Phosphate buffer containing 2.5% glutaraldehyde was added and the samples were fixed for 30 min. The samples were then fixed with 1% osmium tetroxide for 30 min, dehydrated using a concentration series of ethanol and embedded using epoxy resin Epon812. Thin sections were made using a LKB-5 type ultra-thin slicing instrument (LKB Nova, Bromma, Sweden). The slices were stained with uranyl acetate/lead citrate and observed under the H-600 transmission electron microscope (Hitachi, Ltd., Tokyo, Japan).

### Western blot analyses

Cytoplasmic and cell membrane proteins were prepared as described [9]. 5 mL protein extracting liquid A (20 mmol/L Tris HCl (pH 7.5), 0.125 mol/L sucrose, 2 mmol/L EDTA, 015 mmol/L EGTA, 10 mg/L leupeptin, and 50 mmol/L β-mercaptoethanol) containing 1 mmol/L freshly added protease inhibitor mix was added to the cell samples. The samples were dissolved on an ice bath for 10 min and centrifuged at 4°C, 100,000 × g for 1 h. The collected supernatant contained cytoplasmic proteins. 5 mL protein extracting liquid B (enzyme extracting liquid A plus 5% Triton X-100) was added, the samples were stirred at 4°C for 1 h and then centrifuged at 100,000 × g for 1 h at 4°C. The supernatant containing the cell membrane proteins was stored at -80°C until use. Protein quantification was performed using the BCA kit (Bio-rad, Hercules, CA) according to the instructions of the manufacturer.

Total protein (50 μg/lane) was electrophoresed using a PAC300 electrophoresis instrument (Bio-Rad) according to the instructions of the manufacturer. Proteins were then transferred to a cellulose nitrate membrane using an electrophoretic transfer instrument (Bio-Rad). Western blot analyses were performed and the bands visualized using an ECL kit (GE Healthcare, WA). All the required antibodies were purchased from Santa Cruz Biotechnology (Santa Cruz, CA). Films were developed using an X-XQF-2000 gel imaging system (Jianan Photoelectric Ltd., Nanjing, China). Quantitation was done by estimating the optical density of the bands. The β-actin band was used as a normalizing control.

### MMP-9 and uPA immunofluorescent staining

Cells were inoculated into culture wells with a strip-shaped cover slip, incubated for 48 h and rinsed with PBS twice. The cells were fixed with cold acetone for 10 min and stored at -20°C until use. Immunofluorescent staining was performed as follows. The cell climbing slices were soaked in 1% Triton-TBS buffer for 30 min and rinsed with PBS. Samples were blocked with non-immune animal serum at 37°C for 15–20 min and incubated in the diluted first antibody at 4°C overnight. The samples were rinsed with PBS and then incubated in the dark with second antibody labeled with the corresponding fluorescein moiety at 37°C for 2 h. Samples were rinsed with PBS and observed under a Leica DM LB2 fluorescence microscope (Leica, Wetzlar, Germany). For the negative control, the first antibody was replaced by PBS and the remaining procedures and reagents were unchanged. The appearance of red fluorescent areas in the cytoplasm was associated with the presence of MMP-9; the appearance of green fluorescent areas in the cytoplasm was associated with the presence of uPA. Intracellular protein levels were measured using the automatic image analysis instrument of the microscope system. Using the 200× magnified visual field, 100 cells were randomly selected from the upper, lower, left, right, and central zones of the slides in the same experiment. The average optical density (OD) value of the fluorescent particles was measured and the measurement was repeated four times. The data were shown in mean ± standard deviation ( ± *s*).

### Statistical analysis

The experimental data are shown in mean ± standard deviation ( ± *s*). The one-way ANOVA was used for the comparisons among the means. All the analyses were carried out using the SPSS13.0 software (SPSS Inc, Chicago, IL). The significance level was set at P < 0.05.

## Results

### Effects of staurosporine on the cell morphology

**Figure 1:**
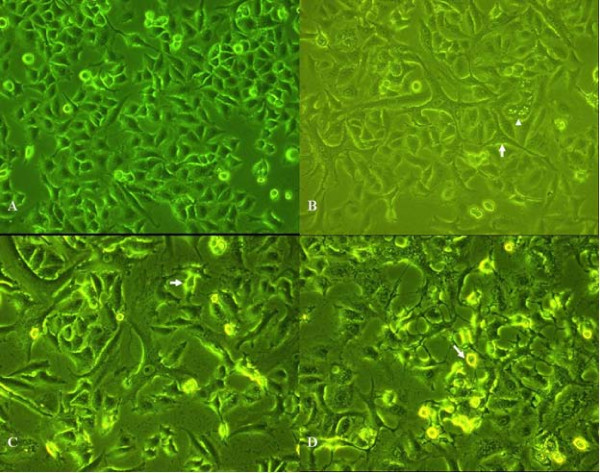
**Effect of staurosporine on the A549 cell morphology**. A: A549 cells in the control group. Cells were short, spindle-shaped and triangle-shaped and resembled epithelial cells (200 ×). B: A549 cells treated with 1 nmol/L staurosporine for 24 h. Some of the cells were long spindle-shaped and exhibit slender protrusions (arrows). Small cavities were visible in the cytoplasm of some cells (arrow heads) (200 ×). C: A549 cells treated with 10 nmol/L staurosporine for 24 h. Some A549 cells retracted and became gradually round-shaped (arrows) (200 ×). D: A549 cells treated with 100 nmol/L staurosporine for 24 h. Some A549 cells retracted, became gradually round-shaped, and shed off (arrows) (200 ×).

Electron microscopy analyses demonstrated that untreated A549 cells were short spindle-shaped and triangle-shaped. They exhibited characteristics resembling those of epithelial cells (Figure 1A). Upon treatment with 1 nmol/L staurosporine for 24 h, morphological changes were observed. A549 cells became long spindle-shaped and narrow protrusions appeared. In addition, some small cavities were observed in the cytoplasm of some cells (Figure 1B). Twenty-four hours after treatment with 10 nmol/L staurosporine, some A549 cells retracted and gradually became round-shaped (Figure 1C). When the staurosporine dose increased to 100 nmol/L, the cells gradually became round-shaped and the number of shedding cells increased after 24 hours of treatment (Figure 1D).

### Effect of staurosporine on A549 cell adhesion

We showed that different concentrations of staurosporine could inhibit the adhesion of the A549 cells to Matrigel, and the inhibition was proportional to the concentration of staurosporine (Table 1). The inhibition rate at a staurosporine concentration of 10 nmol/L and 100 nmol/L reached 50% and 74%, respectively. Compared to that of the untreated cells, this difference was significant (P < 0.01) (Table 1).

**Table 1:** Effect of staurosporine on adhesion of A549 cells ( ± *s*, n = 4)

Group	A-value	Inhibition rate (%)	Compared with the control group, *P*-value
Control group	0.381 ± 0.023	Not applicable	Not applicable
1 nmol/L staurosporine	0.317 ± 0.005	17	0.132
10 nmol/L staurosporine	0.189 ± 0.014	50	<0.001
100 nmol/L staurosporine	0.010 ± 0.013	74	0.000

### Effect of staurosporine A549 cell mobility and invasion

We also demonstrated that staurosporine could inhibit the mobility and invasion capability of the A549 cells in vitro. This effect was dose-dependent (Table 2). There was a significant difference in the mobility and invasion of untreated cells versus cells treated with a staurosporine concentration of 10 nmol/L and 100 nmol/L (P < 0.05) (Table 2).

**Table 2:** Effect of staurosporine on the mobility and invasiveness of A549 cells ( ± *s*, n = 4)

Group	Mobility			Invasiveness		
	
	Cells that penetrate membrane	Mobility inhibition rate	*P*-value	Cells that penetrate membrane	Invasion inhibition rate	*P*-value
Control group	149.00 ± 7.12			74.50 ± 4.20		
1 nmol/L staurosporine	126.50 ± 5.57	15%	0.002	62.75 ± 3.86	16%	0.015
10 nmol/L staurosporine	111.75 ± 7.80	25%	<0.001	46.75 ± 5.91	37%	<0.001
100 nmol/L staurosporine	65.50 ± 5.32	56%	<0.001	34.00 ± 2.94	54%	<0.001

### Effect of staurosporine on cell proliferation and apoptosis

**Figure 2:**
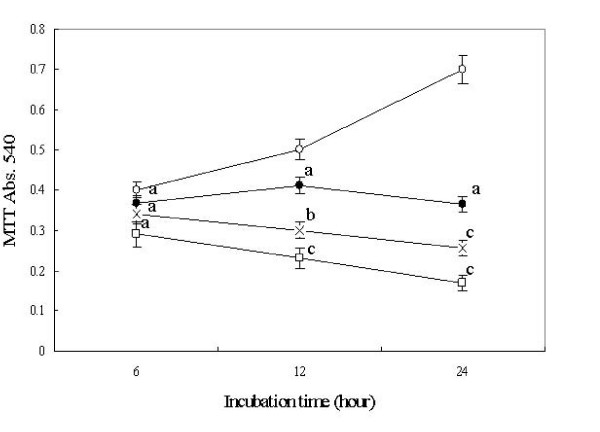
**Effect of staurosporine on cell viability of A549 cells by colormetric MTT assay**. *n *= 4. Mean ± SD. ^a^*P *> 0.05, ^b^*P *< 0.05, ^c^*P *< 0.01 *vs *control. Open circle represents control; solid circle represents 1 nmol/L staurosporine concentration; the cross represents 10 nmol/L staurosporine concentration and the open square represents 100 nmol/L staurosporine concentration

The MTT assay showed that the cell proliferation of A549 cell was significantly inhibited by staurosporine in a dose dependent manner. (Figure 2). FACS assay showed that 6 hours treatment with 100 nM staurosporine caused a significant increase in G_2_/M arrest and apoptosis. The G_2_/M arrest reached its peak at 12 hours treatment and apoptosis reached its peak at 24 hours treatment (Table 3).

**Table 3:** Effect of staurosporine on cell cycle distribution and apoptosis of A549 cells.*n *= 3. Mean ± SD. ^a ^*P *> 0.05, ^c^*P *< 0.01 *vs *control.

Groups/h	Cell cycle/%			Apoptosis/%
		
	G_0_/G_1_	S	G_2_/M	
Control	58.9 ± 1.0	31.2 ± 2.1	9.9 ± 1.2	2.9 ± 0.3
Staurosporine 6 h	58.4 ± 1.9^a^	15.0 ± 0.4^c^	26.6 ± 1.8^c^	7.3 ± 0.4^c^
Staurosporine 12 h	56.0 ± 0.7^a^	9.9 ± 0.4^c^	34.0 ± 1.0^c^	10.6 ± 1.5^c^
Staurosporine 24 h	59.5 ± 1.0^a^	20.7 ± 0.1^c^	19.8 ± 0.9^c^	27.4 ± 0.7^c^

### Ultrastructural changes in A549 cells treated with staurosporine

**Figure 3:**
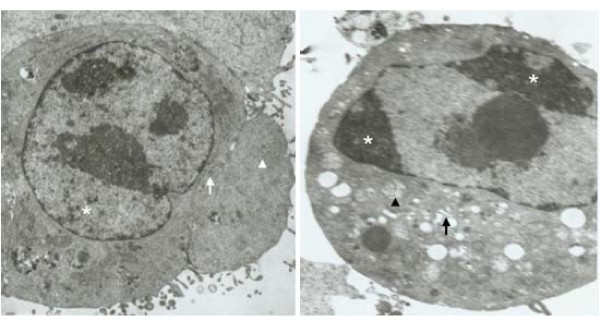
**Ultrastructural changes in A549 cells treated with 100 nM/L staurosporine**. A: Transmission electron microscopy image of an untreated A549 control cell. The nuclear chromatin was homogenized (asterisk), the intracytoplasmic rough endoplasmic reticulum was dense (arrows), and there were a large number of ribosomes (arrow heads). A large number of healthy microvilli were observed at the cell surface (5,000 ×). B: Transmission electron microscopy image of an A549 cell treated with 100 nM staurosporine for 24 h. Typical signs of apoptosis were observed. The nuclear chromatin coagulated to a plate-shaped structure and gathered in the vicinity of the nuclear membrane (asterisk), the cytoplasmic mitochondria were swollen (arrow heads), the endoplasmic reticulum expanded (arrows). The number of microvilli on the cell surface decreased (5,000 ×).

Untreated A549 cells exhibited homogenized chromatin, dense intracytoplasmic rough endoplasmic reticulum, and a large number of ribosomes (Figure 3A). Many microvilli were observed on the cell surface. However, 24 h after treatment with 100 nmol/L staurosporine, the number of microvilli on the cell surface decreased, chromatin coagulation was observed in the nuclei resulting in a plate-like appearance, the nuclear apertures expanded and the cytoplasmic mitochondria were swollen (Figure 3B). The endoplasmic reticulum expanded, and cytoplasmic vacuoles appeared. Vesicles formed that were surrounded by a cell membrane, and some of them contained nuclear debris, which is a typical morphological feature of apoptosis (Figure 3B).

### Effect of staurosporine on the PKC-α level and membrane translocation of the A549 cells

**Figure 4:**
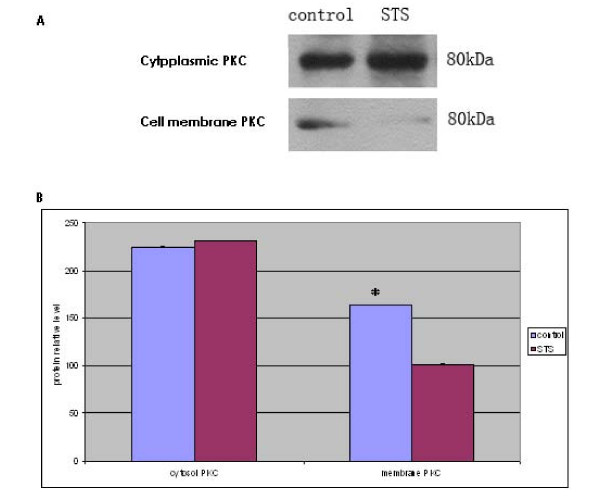
**Effect of staurosporine on membrane localization and level of PKC-α in A549 cells A: representative photo of Western blot**. B: Quantification of densitometry. Comparison with the control group * P

The results of the Western blot analyses showed that although PKC-α was mainly present in the cytoplasm, it was also seen on cell membranes of untreated A549 cells (Figure 4). In A549 cells treated with 100 nM staurosporine for 24 h, the PKC-α level in the cell membrane decreased by 38% (P < 0.01) (Figure 4). No significant differences in the cytoplasmic PKC-α content were observed, suggesting that staurosporine inhibited the activation of PKC-α (Figure 4).

### Effect of staurosporine on the levels of integrin β1, E-cadherin, and LnR

**Figure 5:**
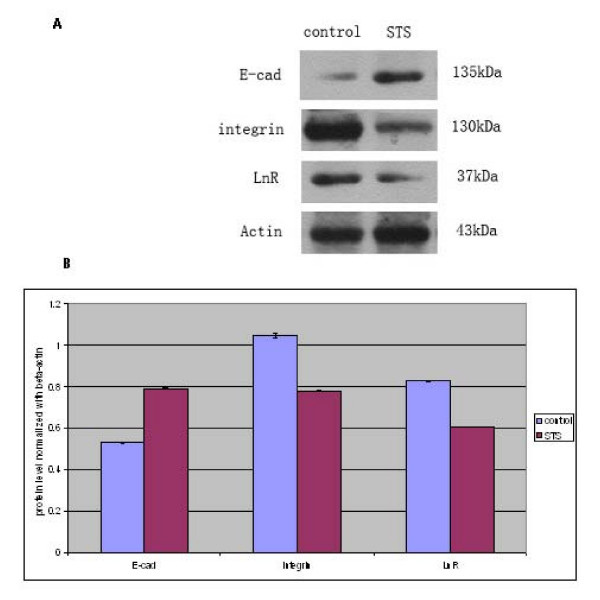
**Effect of staurosporine on the level of integrin β1, E-cadherin, and LnR in A549 cells using Western blot analysis**. A: representative photo of Western blot. B: Quantification of densitometry indicated increased levels of E-cadherin in A549 cells treated with 100 nM stauroporine for 24 h, while integrin β1 and LnR levels were decreased. Intensities are significantly different when P < 0.01.

The Western blot analyses showed that the level of E-cadherin increased and the level of integrin β1 decreased in A549 cells treated with 100 nM staurosporine for 24 h (Figure 5). The protein contents of E-cadherin, integrin β1, and LnR changed by a factor 1.5, 0.74, and 0.73, respectively, compared to that in untreated cells (P < 0.01) (Figure 5).

### Localization of MMP-9 and uPA by immunofluorescence microscopy

**Figure 6:**
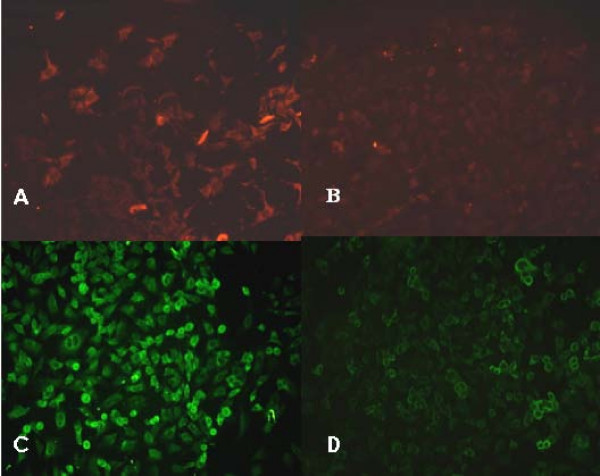
**Effect of staurosporine on the level of MMP-9 and uPA in A549 cells using fluorescence microscopy (× 200)**. A: Control (untreated) A549 cells. The background signal of MMP-9 is relatively high. B: A549 cells treated with 100 nM staurosporine for 24 h. The signal due to MMP-9 decreased. C: Control (untreated) A549 cells. The background signal of uPA is relatively high. D: A549 cells treated with 100 nM staurosporine for 24 h. The signal due to uPA decreased.

The intracytoplasmic level of MMP-9 in untreated A549 cells was relatively high (Figure 6A, Table 4). Image analysis showed a 57.39% decrease (P < 0.01) in the intracytoplasmic fluorescence reflecting MMP-9 levels twenty-four hours after treatment with 100 nM staurosporine (Figure 6B, Table 4). The intracytoplasmic level of uPA in untreated A549 cells was relatively high (Figure 6C, Table 4). However, image analysis showed a 48.37% decrease (P < 0.01) in the intracytoplasmic fluorescence reflecting uPA levels twenty-four hours after treatment with 100 nM staurosporine, (Figure 6D, Table 4).

**Table 4:** Effect of staurosporine on the level of MMP-9 and uPA in A549 cells ( ± *s*, n = 100)

Group	MMP-9		uPA	
	
	OD-value	*P*-value	OD-value	*P*-value
Control group	0.345 ± 0.039		0.521 ± 0.024	
100 nM staurosporine	0.198 ± 0.034	<0.001	0.252 ± 0.038	<0.001

## Discussion

PKC inhibitor staurosporine has become one of the most promising anti-cancer drugs because of its apoptosis promoting effects in tumor cells. This study analyzed the effects of staurosporine on the invasive and metastatic capabilities of lung cancer tumor cells. The results of the cell adhesion experiment in this study showed that staurosporine (100 nmol/L) inhibited the adhesion of A549 cells to Matrigel by 74%. The results of the mobility and invasion experiments showed that staurosporine inhibited the mobility and the invasion of A549 cells by 56% and 54%, respectively. It can be argued that the extent of apoptosis occurring at the highest dose of staurosporine (100 nmol) could have resulted in the lower rates of invasion seen at this dose. However, the lower dose of staurosporine (1 nmol) where there was no significant apoptosis seen, also resulted in a decrease in cell mobility and invasion by 15% and 16% respectively (Figure 2, Table 2).

Tumor invasion and metastasis are hallmarks of malignant tumors and constitute a major cause of ineffective treatment resulting in death of cancer patients. Inhibition of the invasion and metastasis of tumor cells could be a new pathway in the treatment of patients with cancer. Tumor invasion and metastasis is a complex, continuous, multi-step process where metastatic lesions develop in a defined pathway which includes the diffusion of the tumor cells from the primary site, the infiltration of extracellular matrix (ECM), penetration through the vessel walls, intravascular aggregation, and adhesion to the vascular endothelium. All of these processes are related to the adhesion, mobility, and the invasive characteristics of the tumor cells.

Dumont et al[11] reported that the PKC activator phorbol 12-myristate 13-acetate (PMA) could promote tumor metastasis in animal models, and PKC inhibitors could inhibit the tumor metastasis. Haier et al. [12] found that PMA led to increased adhesion of colon cancer cells to type I collagen, but PKC inhibitors brought about a decrease in this adhesion. Furthermore, it was also shown that PMA could decrease the homogeneity adhesion (the adhesion between tumor cells) of the colorectal cancer HT299 cells and increase their heterogeneity adhesion (adhesion between tumor cells and the extracellular matrix) to HUVEC3, but PKC inhibitors could antagonize the above effects [13].

Staurosporine is a potent inhibitor of PKC and most other kinases (including the tyrosine protein kinases). Here, we demonstrate that the PKC-α content in the A549 cells decreased to 22.73% of that in the control group 24 h after treatment with 100 nM staurosporine (P < 0.01), while the cytoplasmic PKC-α content did not change significantly. This suggests that staurosporine can inhibit PKC-α activation of the A549 cells and is consistent with its effect on the cell adhesion, mobility, and invasive capability of A549 cells.

The increased heterogeneity adhesion between the tumor cells and the vascular endothelial cells and the extracellular matrix is the basis for the mobility and invasion and metastasis of the tumor cells. Most adhesion receptors are substrates of PKC. A change in receptor configuration achieved via PKC-mediated receptor phosphorylation affects receptor-ligand binding properties, ultimately regulating the adhesion function. To further explore the potential mechanisms of staurosporine-mediated inhibition of cell adhesion in A549 cells, we examined the changes in adhesion molecules, E-cadherin, LnR, and integrin β1. E-Cadherin is a calcium-dependent adhesion molecule mediating the homogeneity adhesion between cells, which exists widely in various types of epithelial cells. It can form complexes with intracytoplasmic soluble protein catenins. Our results show that the E-cadherin level increased 2.91 times in A549 cells 24 h after treatment with 100 nM staurosporine, suggesting that staurosporine can promote the homogeneity adhesion between tumor cells.

Laminin (Ln) is the main component of the basement membrane. The laminin receptor (LnR) on the surface of the tumor cell can interact with the Ln in the basement membrane. A large number of in-vitro experiments previously confirmed that the levels of the LnR on the surface of tumor cells were closely related to heterogeneity adhesion and mobility [14,15]. This study showed that the LnR protein content in the A549 cells decreased to 57% of that in the control group 24 hours after treatment with 100 nM staurosporine.

Our results also show that staurosporine inhibited integrin β1 expression. Integrin β1 primarily mediates adhesion between the cells and the extracellular matrix. Its ligands are collagen, fibronectin, laminin, and fibrinogen in the extracellular matrix. It forms "ligand-integrin-transmembrane cytoskeleton system" local adhesion devices. PKC may activate the adhesion of cells through phosphorylation of integrin, increasing integrin levels on the cell surface, reconstruction of the cytoskeleton, or activation of transcription factors [16].

The integrity of the basement membrane is a key component affecting the invasion and metastasis of the tumor. MMP and uPA are proteases that participate in basement membrane destruction; they play an important role in the invasion and metastasis of the tumor. MMP-9 (92 kD gelatinase B) is present in many cells, including endothelial cells, fibroblasts, osteoblasts, cartilage cells, and invasive tumor cells. PKC agonist PMA can regulate the expression of MMPs, TIMPs, uPA receptor, and uPA inhibitor PAI-1 [17-19] The immunofluorescence results of this study showed that a 24-hour treatment with 100 nM staurosporine resulted in 57.39% and 48.37% decreases in the level of MMP-9 and uPA respectively in A549 cells. This indicated that staurosporine can decrease the invasive capability of cancer cells through the inhibition of MMP-9 and uPA protein levels in lung cancer cells and is consistent with other reports [20-22] We understand the importance of interpreting these results in the light of the increased apoptosis observed in staurosporine treated cells and the resulting loss in cell viability. Additionally, realizing that staurosporine is a non-specific PKC inhibitor, our future work will include assessing the role of more specific PKC inhibitors on cell adhesion and mobility in A549 cells.

## Conclusion

In summary, this study illustrates that staurosporine inhibits cell adhesion, mobility, and invasion of A549 cells. These effects could be mediated through the inhibition of PKC-α, induction of E-Cad expression, and decreased integrin β1, LnR, MMP-9 and uPA levels. It is worth noting that staurosporine is a non-specific PKC inhibitor with multiple biological effects. Therefore, additional studies are required to study the effect of staurosporine in other cell lines. Our future goals include decoding the relationship between the various proteins tested herein and the staurosporine-mediated decreases in adhesion, mobility, and invasiveness of A549 lung cancer cells. Nevertheless, our results represent an important step forward in understanding the development of lung carcinoma and designing novel strategies to inhibit metastasis of the tumor by targeting the adhesion, mobility and invasive properties of these tumor cells.

## Competing interests

The authors declare that they have no competing interests.

## Authors' contributions

YW participated in the study design, statistical analysis, and manuscript preparation and drafting. HY, HL, JH and XS participated in the study design. All authors read and approved the final manuscript.

## Pre-publication history

The pre-publication history for this paper can be accessed here:

http://www.biomedcentral.com/1471-2407/9/174/prepub
